# Efficiency of Fluid Treatments with Different Sodium Concentration in Children with Type 1 Diabetic Ketoacidosis

**DOI:** 10.4274/jcrpe.v3i3.29

**Published:** 2011-09-09

**Authors:** Şenay Savaş-Erdeve, Merih Berberoğlu, Pembe Oygar, Zeynep Şıklar, Tanıl Kendirli

**Affiliations:** 1 Ankara University School of Medicine, Department of Pediatrics, Division of Pediatric Endocrinology, Ankara, Turkey; 2 Ankara University School of Medicine, Department of Pediatrics, Pediatric Intensive Care Unit, Ankara, Turkey; +90 505 481 21 50 senaysavas@yahoo.com

**Keywords:** diabetic ketoacidosis, sodium concentration, rehydration fluid, effective plasma osmolality

## Abstract

**Objective:** The management of children with diabetic ketoacidosis (DKA) continues to be a controversial issue with regard to amount of intravenous fluid to be given, rate of delivery of fluid, and type of fluid to be used. We aimed to analyze the results obtained by administration of rehydration fluids of  two different sodium (Na) concentrations (75 mEq/L vs. 100 mEq/L ) in the treatment of children with DKA.

**Methods:**  Thirty-two children with DKA were assessed for efficacy and safety of fluid treatment. After an initial rehydration time, intravenous fluids were switched to a 5% dextrose solution with a Na content of 75 mEq/L (Group I, n=19) or 100 mEq/L (Group II, n=13). Venous blood samples were collected from all subjects at diagnosis and at the 4th,  8th, 16th and  24th hours of treatment.

**Results:** Changes in blood glucose levels did not differ significantly between the two  groups at the 4th,  8th, 16th and  24th hours of the follow-up. Nadir effective plasma osmolality (Peff osm) and Peff osm levels also did not show statistically significant differences. Plasma sodium (PNa) level did not drop lower than the level at diagnosis in both groups. The changes in PNa concentrations in the two groups were not statistically significant at diagnosis or in follow-up samples (p=0.74). pH, anion gap, pCO2 and HCO3 levels were also similar in Group I and Group II. The duration of a pH level of <7.3 was shorter in Group II,   but this was not statistically significant (p=0.65). None of the patients enrolled in this study developed cerebral edema.

**Conclusion:** The efficacy and safety of rehydration fluids with Na concentrations of 75 or 100 mEq/L  did not reveal any differences in children with DKA.

**Conflict of interest:**None declared.

## INTRODUCTION

Diabetic ketoacidosis (DKA) in children is a serious acute complication of diabetes mellitus and continues to be an important cause of morbidity and mortality. Treatment of children with DKA is a complex blend of emergency therapy and underlying disease management (1). Immediate  but cautious fluid therapy is an essential component in the management of patients with DKA. The European Society for Paediatric Endocrinology (ESPE) and other international consensus recommendations have emphasized the  importance of  appropriate sodium (Na) concentration in the rehydration solutions used in treatment of DKA (2,3,4,5). Initially, 0.9% isotonic salt solution ([Na]= 154 mEq/L) has been considered the most suitable parenteral solution to initiate rehydration for the first hour or in the first 4 hours, depending on different authors, and thereafter is switched to a solution of lower tonicity  (≥0.45% saline solution; ([Na]≥77 mEq/L) until complete rehydration (2,3,4,5). However, there continues to be a debate about the amount of intravenous (IV) fluid to be given, the speed of delivery of fluid, and the type of fluid to be used. Because a 0.45% saline solution is not available in Turkey, treatment of DKA patients is initiated with 0.9% Na saline and continued by a mixture of 5% dextrose and 0.9% Na saline. In the past, a solution with a Na concentration of 75 mEq/L has been used. However, in our clinic we observed that some patients developed low plasma sodium levels (PNa) (corrected plasma Na <130 mEq/L) on the 8th hour of treatment with this kind of approach. Considering that this treatment regimen might be a risk factor for development of cerebral edema (CE), we began to use a solution with a Na concentration of 100 mEq/L in the treatment  of our DKA patients.

   In this study, we aimed to analyze the influence of  two solutions with different Na concentrations in the  rehydration of children with DKA.  

## MATERIALS AND METHODS

Patients who received an IV solution with a Na  concentration of 75 mEq/L constituted Group I. This group consisted of patients who were treated before year 2006.  Patients who received a solution with a Na concentration of 100 mEq/L constituted Group II.  All data including age, sex, and new-onset diabetes were retrospectively collected from patient records. The study was approved by the local Ethics Committee.

 This study included patients younger than 18 years of age who were admitted to the pediatric intensive care unit from 2002 to 2009. DKA was defined as having a glycemia >200 mg/dL (11.4 mmol/L), a venous pH <7.30 or a plasma bicarbonate level <15 mmol/L, and  ketonuria (2). Effective plasma osmolality (Peff osm) was calculated as 2 x PNa+ PGlucose (plasma glucose) in mmol/L (6). PNa was corrected for hyperglycemia using the method of Katz (7), whereby corrected plasma sodium (PNacorr) (mmol/L)=actual Na+ [(serum glucose mmol/L - 5.6)/5.6] x 1.6. Definition of  cerebral edema (CE) was made according to the criteria defined by Edge et al (1). 

 In both groups, initial rehydration was performed with isotonic solutions in the first hour of treatment. The total volume to be given was calculated assuming a 10% deficit plus maintenance fluid. Amounts of fluids used in the  initial resuscitation were subtracted from the total volume calculated for 48 hours and the infusion rate was adjusted accordingly. After initial rehydration, IV fluids were switched to solutions containing 5% dextrose and [Na+] 75 mEq/L (Group I) or [Na+] 100 mEq/L (Group II). The only source of Na used in our patients was Na chloride (NaCl). The patients in Group I had received IV fluids with a Na  concentration of 75 mEq/L (1/2 isotonic NaCl plus 1/2 5%  dextrose) and those in Group II received IV fluids with a Na concentration of 100 mEq/L (2/3 isotonic NaCl plus 1/3 5% dextrose). During rehydration, the potassium concentration of the IV fluids was adjusted as 40 mEq/L. The patients were started on oral intake and subcutaneous insulin as soon as the acidosis was resolved, serum Na level became stable, and vomiting had stopped. After transition to oral intake, the amount of oral fluid was subtracted from the ongoing IV fluid treatment. 

 Data on age, sex, date of onset and duration of diabetes at the time of diagnosis were recorded. Data pertaining to the same patient in different DKA episodes were recorded separately. After admission, blood glucose was measured at hourly intervals. Samples of venous blood for blood gases and electrolytes were taken at admission and at  the 4th, 8th, 16th and 24th hours after admission. 

 The Mann-Whitney U test was used to compare the clinical characteristics and laboratory values between the two groups. ANOVA test was used for variance analysis between groups. A p-value below 0.05 was accepted as significant.

## RESULTS

A total of 32 children who received DKA treatment were enrolled in the study. Twenty-six of these patients had  new-onset diabetes. Group I included 19 patients and 13 patients were in Group II. None of the patients enrolled in this study developed CE. Clinical characteristics and  biochemical data for the two groups at the time of  admission are given in Table 1. 

**Figure 1:**
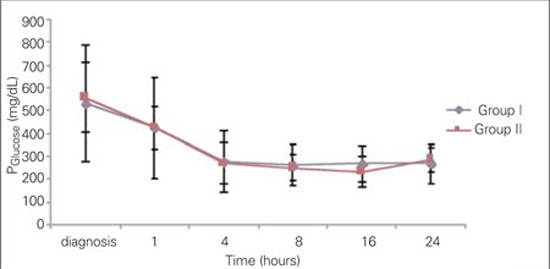
Figure 1. Time courses for PGlucose from the start of therapy up to 24 hours of therapy PGlucose: mg/dLd

**Figure 2:**
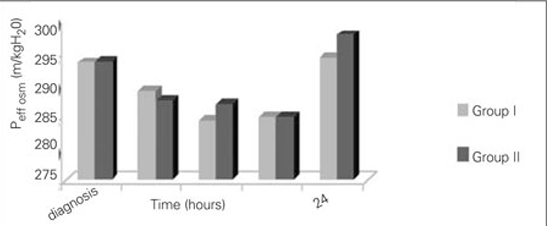
Figure 2. Time courses for Peff osm from the start of therapy up to 24 hours of therapy Peff osm: mOsm/kgH20

**Figure 3:**
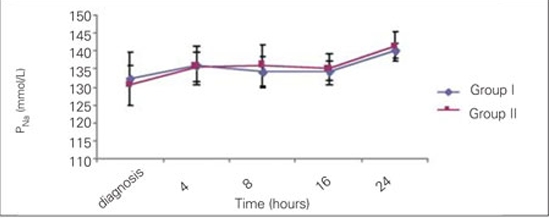
Figure 3. Time courses for PNa from the start of therapy up to 24 hours of therapy PNa (measured sodium): mmol/L

**Figure 4:**
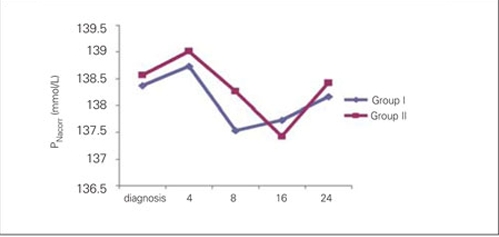
Figure 4. Time courses for PNacorr from the start of therapy up to 24 hours of therapy PNacorr (corrected sodium): mmol/L

The courses of glycemia and effective osmolality in our patients are shown in Figure 1 and Figure 2. Blood glucose levels after one hour of rehydration  were not statistically different between groups (424.1±223.3 mg/dL in Group I and 424±96 mg/dL in Group II) (p=0.236). The most 

significant decrease in blood glucose was noted at the 4th hour of treatment in both groups and the proportions of change (in percentages) in Group I and Group II were as  44.8±14% and 50.4±15%, respectively. Time at which  nadir blood glucose level was reached was the 8th hour in Group I and the 16th hour in Group II. Proportion of change (in percentages) in blood glucose levels did not differ significantly between the groups at 4th,  8th, 16th and  24th hours. Duration of insulin administration by IV infusion showed a significant difference between the groups, and was 5  (2-62) hours in Group I and 23 (2-74) hours in Group II (p=0.018).  The median transition time to oral intake in Group I  was 8 (4-62) hours. This period  was shorter as compared to Group II [23 (3-74) hours] (p=0.033).  The Peff osm dropped during the first 8 hours of  treatment in Group I, whereas in Group II, such fall occurred at the 16th hour of therapy . Nadir Peff osm level was  similar in Group I and Group II (Figure 2). The difference in Peff osm between the two groups did not show statistical significance during follow-up (p=0.80).  The course of natremia in our patients is shown in Figure 3. The PNa level did not drop lower than the level at diagnosis in either group. Mean±SD of PNa levels at diagnosis and  during follow-up are shown in Table 2. The difference in PNa between Group I and Group II was not statistically significant at either the diagnosis or the 4th, 8th, 16th and 24th hours after diagnosis (p=0.35). The rate of patients with PNa  concentrations lower than 135 mmol/L was similar in both groups at the 4th (46.2%; 38.5%), 8th (40%; 53.8%), 16th (53.8%; 38.5%) and 24th (0%) hours after diagnosis (p>0.05). A PNa concentration higher than 145 mmol/L was detected in only one patient of each group at the 24th hour after diagnosis (p>0.05). The course of PNacorr in our patients is shown in Figure 4. The difference between the groups regarding PNacorr was not statistically significant at diagnosis and during  follow-up (p=0.74) (Table 2). The ratio of patients with a PNacorr concentration lower than  135 mmol/L was also similar in both groups (p>0.05). The course of serum potassium levels in our patients is shown in Table 2. There was no hypokalemia either in the beginning or during the follow-up in either group.  pH, anion gap, pCO2 and HCO3 levels were also similar between Group I and Group II at diagnosis and at  follow-up (Table 2). Anion gap dropped below 20 mEq/L at the 16th hour in both groups. The duration of a low pH (>7.3) in Group II  was shorter than in Group I, but this was not statistically significant (13.23±10.11 hours in Group II, 20.47±21.76 hours in Group I) (p=0.65). 

**Table 1:**
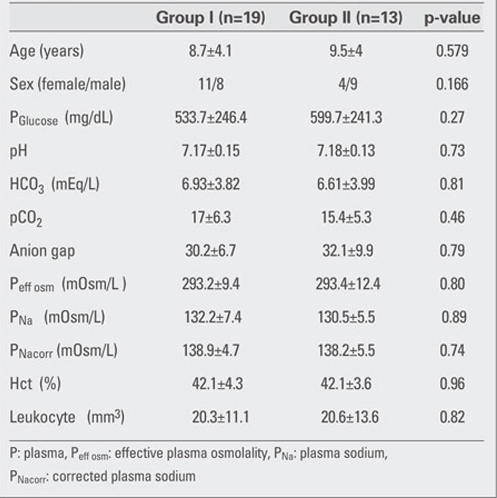
Table 1. Clinical and biochemical findings in the two groups at the time of admission

**Table 2:**
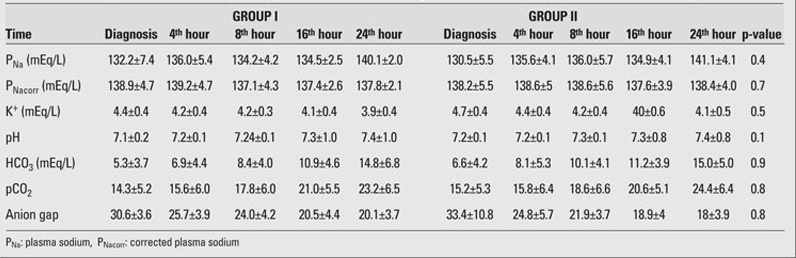
Table 2. The PNa, PNacorr, K+, pH, HCO3, pCO2, anion gap in the two groups at diagnosis and at the 4th, 8th, 16th and 24th hour after diagnosis

## DISCUSSION

In most countries, treatment of DKA follows standard protocols. In spite of meticulous treatment, a few children develop complications, of which CE is the most dreaded.  CE typically occurs 4-12 hours after the initiation of  treatment for DKA, but may develop at any time during treatment (8,9). The cause or causes of CE remain unknown. The use of hypotonic fluids during resuscitation has been  suggested as a cause (10,11), as have reductions in PNa  concentration 10,11,12) and rapid changes in plasma  osmolality (13,14). In the light of current knowledge,  hyponatremia should be avoided in children with DKA (15,16). Rother et al (17) investigated whether rehydration of young patients with DKA by use of a solution with 75 mmol/L of Na would be associated with a decline in serum  concentrations and they found that such rehydration did not lead to decline in the uncorrected serum Na level. There is still insufficient evidence correlating the Na concentration in the rehydration solutions with the PNa levels during  treatment (2,18). A tendency to increase Na levels during rehydration has been associated with a favorable outcome and lesser incidence of CE. However, an important concern  regarding the use of isotonic instead of hypotonic solutions for rehydration is the risk of triggering hypernatremia (9,19,20). To our knowledge, there is only one reported study comparing hypotonic and isotonic solutions in DKA treatment. The results of this study have suggested that the amount of Na in  rehydration fluids is a relevant and independent factor that favors the acquisition of a positive tendency of natremia (21). These authors have stated that a higher PNa level is reached with the use of an isotonic perfusate during rehydration than that achieved with a perfusate with a Na content in a lower range as recommended by international guidelines (2,3,4).  In contrast to the results reported by Toledo et al (21), in our study, the course of PNa, PNacorr and Peff osm at  follow-up was not found to show any difference between patients receiving rehydration solutions containing 75 mEq/L  and 100 mEq/L of Na. Also in contrast to the results reported by these same authors,  higher Na levels or  hypernatremia were not observed in the patients receiving anisotonic solution. The ratio of patients with PNa and PNacorr concentrations greater than 135 mEq/L during the follow-up were similar and, PNa and PNacorr  values did not drop lower than diagnosis levels in both groups. 

 In DKA, the hyperglycemia should be reduced gradually at a rate of 50 to 100 mg/hr. Early switching to a dextrose-containing solution, at a blood glucose level of about 250 mg/dL, helps to avoid rapid fluctuations in osmolality (22). In our treatment protocol, to obtain a 0.45% saline solution, we added 5% dextrose to the IV fluid after one-hour  rehydration. We think that this prevents fluctuations in  plasma osmolality and plays a major role in protecting from CE. However, there is a need for further studies to clarify the efficiency of this regimen in protecting from  CE. 

 Another important aspect is the correlation between Na administration and time needed to normalize acidosis. Several studies have suggested that the use of fluids with higher Na content would more rapidly correct acidosis (23). The duration of pH<7.3 in Group II was shorter than that in Group I but statistically not significant. In our study, the use of an isotonic solution did not create a difference in anion gap, HCO3 or pCO2 levels. However, longer intravenous insulin duration and delay in passage to subcutaneous insulin treatment might be due to variable individual responses.  We conclude that the efficiency of rehydration fluids with a Na concentration of 75 or 100 mmol/L in children with DKA did not differ the course of PNa, PNacorr and  Peff osm. This is the second trial that investigated the  comparative efficiency of IV solutions with 75 and 100 mEq/L Na concentrations in the treatment of DKA. Thus, although this study may serve to fill a gap in this subject, it had some limitations. Firstly, we used a small sample size and retrospective design and, secondly, we did not observe any patient who developed CE in our sample. Therefore, we believe that it will be logical to prospectively investigate  the fluid concentrations used in the treatment of DKA patients who develop CE and their effect, if any, on the  development  of this condition. 
